# Advanced Mathematical Methods in Dental Bioengineering and Biomaterials Machining

**DOI:** 10.3390/biomimetics11070448

**Published:** 2026-06-29

**Authors:** Ján Duplák, Dušan Knežo

**Affiliations:** Faculty of Manufacturing Technologies with a Seat in Presov, Technical University of Kosice, Bayerova 1, 080 01 Presov, Slovakia; dusan.knezo@tuke.sk

**Keywords:** dental bioengineering, numerical simulation, biomaterials, machining

## Abstract

This article presents a systematic analysis of the application of advanced mathematical and computational approaches in dental bioengineering, with a focus on biomaterials processing and machining-related technologies. The aim is to critically synthesize current knowledge on the use of numerical simulations, statistical modeling, and algorithm-based methods in the analysis and optimization of technological processes in dentistry. The review was conducted following the PRISMA framework to ensure a transparent and reproducible selection of relevant studies addressing the intersection of dental applications, manufacturing processes, and computational modeling. The results reveal that the current research does not constitute a unified modeling framework, but rather a heterogeneous set of approaches targeting specific aspects of biomaterial processing. The analyzed studies demonstrate the application of finite element analysis, empirical statistical models, and geometry-based computational methods, particularly in processes such as drilling and grinding of ceramic dental materials. These approaches enable detailed analysis of mechanical and thermal loading conditions, as well as partial optimization of process parameters. However, their applicability is often limited by their empirical nature, lack of integration, and insufficient linkage to real-time process control. The synthesis highlights a significant research gap in the development of integrated and multiphysics modeling frameworks capable of combining mechanical, thermal, and geometrical aspects of machining processes. Future research should focus on the implementation of digital twins, adaptive process control, and personalized modeling strategies to enhance the accuracy, efficiency, and predictability of dental biomaterial processing.

## 1. Introduction

Current research in dental bioengineering is characterized by the extensive application of mathematical modeling, numerical simulations, and optimization algorithms in the analysis of biomaterials and implant systems. The finite element method (FEA) remains the dominant analytical tool, evolving from early studies of axial and non-axial loading [1] to advanced simulations of stress distribution, fatigue behavior, and implant–bone interaction [2,3]. Despite the high predictive capability of these approaches, significant challenges persist, particularly in defining realistic boundary conditions at the bone–implant interface [4] and in modeling complex three-dimensional systems [5,6,7].

**Figure 1:**
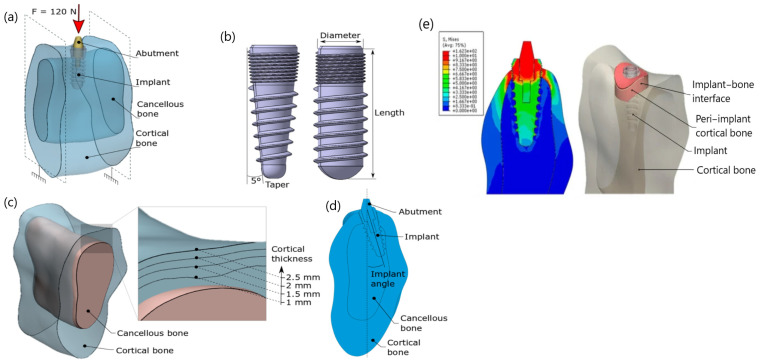
Representative schematic of finite element modeling of a dental implant system. (**a**) Implant–bone model with applied loading conditions; (**b**) geometric characteristics of the implant; (**c**) structure of cortical and cancellous bone; (**d**) implant positioning and angulation; (**e**) example of stress distribution obtained using finite element analysis. Adapted from Didier et al. [8] under the Creative Commons CC BY 4.0 license.

Figure 1 illustrates a typical finite element modeling framework of a dental implant system, including geometric representation, loading conditions, and stress distribution within the implant–bone interface. These modeling approaches typically involve the definition of geometry, material properties, and loading conditions, followed by numerical evaluation of stress distribution.

To reduce computational cost and improve simulation efficiency, statistical and optimization-based methods are increasingly employed, including response surface methodology and fractional factorial design [9], as well as surrogate models based on Kriging approximation [10]. These approaches enable efficient exploration of parameter interactions and optimization of implant design with respect to mechanical performance and fatigue resistance [11]. In parallel, advanced algorithmic techniques such as artificial intelligence, genetic algorithms, and multi-objective optimization are being widely adopted [12,13], allowing simultaneous optimization of multiple design criteria.

Significant attention has also been devoted to the development of advanced biomaterials and structural architectures, including functionally graded materials (FGMs) and porous biomimetic structures. These systems are designed using metaheuristic optimization approaches [14,15] and analyzed through numerical simulations focusing on stress distribution and mechanical stability [16,17,18]. Topology optimization and lattice design strategies, such as triply periodic minimal surfaces (TPMS) and Voronoi-based architectures, have demonstrated significant potential for improving load transfer and biomechanical response [16,17].

The dynamic behavior of implant systems is further influenced by time-dependent biological processes, which are increasingly incorporated into computational models. These include simulations of bone remodeling [19,20], optimization of material compositions [21], and modeling of implant–tissue interactions [22]. To enhance model robustness, stochastic approaches such as generalized polynomial chaos [23] and probabilistic risk assessment models [24] are also applied.

Beyond biomechanical aspects, mathematical modeling is used to analyze thermal phenomena and material-related factors affecting osseointegration and long-term implant stability [25,26,27]. At the same time, new material concepts, including nanocomposites and advanced ceramic systems, are being developed to enhance biological performance and mechanical compatibility [28,29].

Recent trends in dental implantology further emphasize the development of advanced implant materials and designs, including titanium and titanium alloys, zirconia, titanium–zirconium alloys, porous structures, functionally graded materials, and biomimetic lattice architectures [16,17,18]. These trends are closely linked to surface modification and digital design strategies aimed at improving osseointegration, wettability, mechanical reliability, and long-term functional performance [30,31,32]. From a processing perspective, current research increasingly highlights the need to relate material selection, surface topography, manufacturing route, and process parameters such as rotational speed, feed rate, cutting or drilling forces, temperature rise, material removal rate, and surface roughness [32].

Despite the extensive development of mathematical and computational approaches, most existing studies primarily focus on biomechanical analysis and implant–tissue interaction. In contrast, significantly less attention has been given to manufacturing processes, particularly machining operations such as milling, drilling, and grinding, and their integration with mathematical modeling. This imbalance leads to a fragmentation of knowledge, where process-related and material-related aspects are often investigated separately.

Recent developments also indicate a growing integration of digital technologies, including CAD (Computer-Aided Design)/CAM (Computer-Aided Manufacturing) systems, artificial intelligence, and digitally guided surgical workflows [30,31,32], aiming to establish comprehensive digital pipelines. However, a systematic linkage between manufacturing process modeling, biomaterial design, and clinical application remains insufficient.

Based on these considerations, there is a clear need for a systematic evaluation of mathematical and computational approaches with a focus on their application in dental biomaterials processing and manufacturing technologies. Therefore, the aim of this study is to analyze and critically synthesize current knowledge on the use of numerical, statistical, and algorithm-based methods in the context of machining processes in dental bioengineering. The systematic review was conducted following the PRISMA (Preferred Reporting Items for Systematic Reviews and Meta-Analyses) methodology, with an emphasis on identifying studies that integrate dental applications, manufacturing processes, and mathematical modeling.

## 2. Materials and Methods

Table 1 summarizes the literature search strategy used to identify relevant publications for this review. The PRISMA framework was applied as a methodological guideline to ensure transparency and reproducibility of the literature identification and screening process, while the final synthesis was conducted in a narrative form. This approach was selected because it supports structured reporting, explicit inclusion and exclusion criteria, and a clear presentation of the study selection process, although its limitation is that it primarily guides reporting and study selection rather than the interpretation of heterogeneous findings. The identification, screening, and selection stages were defined in advance to ensure that the retrieved studies were evaluated consistently according to the same eligibility criteria. This structured procedure reduced the risk of selection bias by applying explicit inclusion and exclusion criteria at each screening stage and by documenting the reasons for exclusion in the PRISMA flow diagram. The completed PRISMA checklist is included in the Supplementary Files. The review protocol was retrospectively registered in the Open Science Framework (OSF) Registries during the peer-review process. Scopus and Web of Science Core Collection (WoS) databases were searched to identify studies relevant to the topic from database inception to the date of the search, with an English-language restriction.

The search strategy was constructed using three groups of keywords focused on (A) dental bioengineering and dental biomaterials, (B) manufacturing and machining processes, and (C) mathematical modeling and computational methods. The first group included terms related to dental applications, such as dental bioengineering, dental biomaterials, oral biomaterials, dentistry, prosthodontics, and implantology. The second group covered technological processes, including machining, manufacturing, processing, milling, drilling, grinding, CAD/CAM, additive manufacturing, and 3D printing. The third group focused on mathematical and computational approaches, including mathematical models, computational models, simulation, finite element analysis (FEA/FEM—Finite Element Method), optimization, numerical analysis, algorithms, and predictive modeling.

The individual keyword groups were combined using Boolean operators to ensure that only studies containing relevant terms from all three areas (A and B and C) were included in the results (see Table 1).

The studies were included based on the following criteria: (1) publications available in the English language; (2) articles published in peer-reviewed scientific sources, including journals and conference proceedings; (3) research related to dental bioengineering, dental biomaterials, or oral biomaterials; (4) studies addressing manufacturing or processing methods applied in dentistry, such as machining operations (milling, drilling, grinding), as well as CAD/CAM technologies, where directly linked to material processing or machining-related workflows; (5) studies employing mathematical or computational techniques, including finite element analysis (FEA/FEM), numerical simulations, statistical approaches, optimization methods, or algorithm-based models; and (6) studies combining computational modeling with technological or manufacturing processes in dental applications.

The studies were excluded based on the following criteria: (1) publications not classified as peer-reviewed scientific outputs, such as books, book chapters, review articles, or non-scientific documents; (2) studies outside the scope of dental bioengineering or not related to dental or oral biomaterials; (3) studies focused on educational activities, e-learning systems, surveys, or bibliometric analyses without a direct connection to engineering or material processes; (4) studies lacking the application of mathematical or computational methods in relation to manufacturing or processing contexts; and (5) studies lacking an integrated focus on dental applications, manufacturing or processing techniques, and numerical or computational methods, including modeling, simulation, or optimization. The review was conducted based on the PICO (Population, Intervention, Comparison and Outcome) criteria:

- P (Population): Dental biomaterials and prosthetic components (ceramics, zirconia, implant materials, dental crowns, implant-supported systems);
- I (Intervention): Application of advanced mathematical and computational methods, including numerical simulation, finite element analysis (FEA/FEM), statistical modeling, and optimization techniques in manufacturing and machining processes (e.g., grinding, drilling, CAD/CAM fabrication);
- C (Comparison): Comparison of different modeling approaches and manufacturing strategies, including conventional versus optimized processes, and analytical versus numerical methods;
- O (Outcome): Improvement in process efficiency and material performance, including machining accuracy, reduction in thermal and mechanical loads, optimization of process parameters, improved process predictability, and enhanced mechanical properties and reliability of dental biomaterials.

## 3. Results

A total of 1901 records were identified after the removal of duplicate publications (*n* = 460) using reference management software Zotero. These records were subsequently subjected to an initial screening based on title and abstract (Screening A), with all inclusion and exclusion decisions verified by the authors.

During Screening A, a total of 1453 publications were excluded. Of these, 169 records were removed based on publication type, including books, book chapters, and review articles. Review papers were identified through explicit labeling (e.g., review, systematic review, meta-analysis) in the title or abstract and were excluded to ensure that only primary research studies were considered. An additional 49 publications were excluded as they were not relevant to the study scope, including fields such as geology, civil engineering, and food science. Furthermore, 356 studies were excluded due to the absence of a medical or dental context. The largest group of excluded records (879 studies) consisted of publications related to the medical field but lacking any connection to manufacturing or material processing.

After the initial screening, 448 publications remained eligible and were included in the next stage (Screening B). In this phase, a more detailed evaluation was performed, focusing on the relationship between dental applications and manufacturing processes. A total of 320 publications were excluded. Among these, 42 records could not be assessed due to the unavailability of the full text. Additionally, 46 studies were excluded as they focused on other medical domains, such as oncology, general orthopedics, or soft tissue research, without direct relevance to dental applications. The majority of excluded studies (232 publications) contained keywords related to machining; however, a detailed content analysis revealed that their primary focus was not on manufacturing processes such as milling, drilling, tool wear, or CAD/CAM technologies, but rather on peripheral or unrelated aspects.

A total of 128 publications advanced to the final screening stage (Screening C). At this stage, strict inclusion criteria were applied, requiring the simultaneous presence of dental relevance, manufacturing processes, and mathematical or computational modeling. Out of these, 122 studies were excluded. The largest group (*n* = 58) consisted of publications that did not include mathematical or simulation-based modeling of machining processes and were primarily based on experimental or clinical measurements. A further 42 studies (*n* = 42) were excluded because, although they employed numerical methods (e.g., FEM/FEA), their primary focus was on biomechanical analysis (e.g., masticatory forces, fatigue behavior) rather than manufacturing process optimization. Another group (*n* = 17) were excluded due to their focus on additive manufacturing technologies (e.g., 3D printing), which did not meet the criteria for subtractive machining processes. The remaining five studies (*n* = 5) were excluded as they addressed educational systems, IT-based applications, or bibliometric analyses without a direct connection to physical or mathematical modeling of manufacturing processes.

After applying all selection criteria, a total of 6 studies were included in the final qualitative analysis.

The relatively low number of included studies reflects the highly specific scope of this review, which requires the integration of dental applications, manufacturing (machining) processes, and advanced mathematical or computational methods. A substantial number of initially identified publications were excluded due to the absence of one or more of these key components, most commonly due to the lack of computational modeling or insufficient relevance to manufacturing processes.

The final set of studies encompasses a range of mathematical and computational approaches, including statistical modeling (DOE—Design of Experiments, RSM—Response Surface Methodology), numerical simulations (FEM), algorithm-based modeling, and computer-assisted simulation systems. One of the included studies represents an engineering design-oriented approach focused on surface optimization of zirconia, rather than classical mathematical modeling; however, it was included due to its direct relevance to material processing and applicability in dental manufacturing.

**Figure 2:**
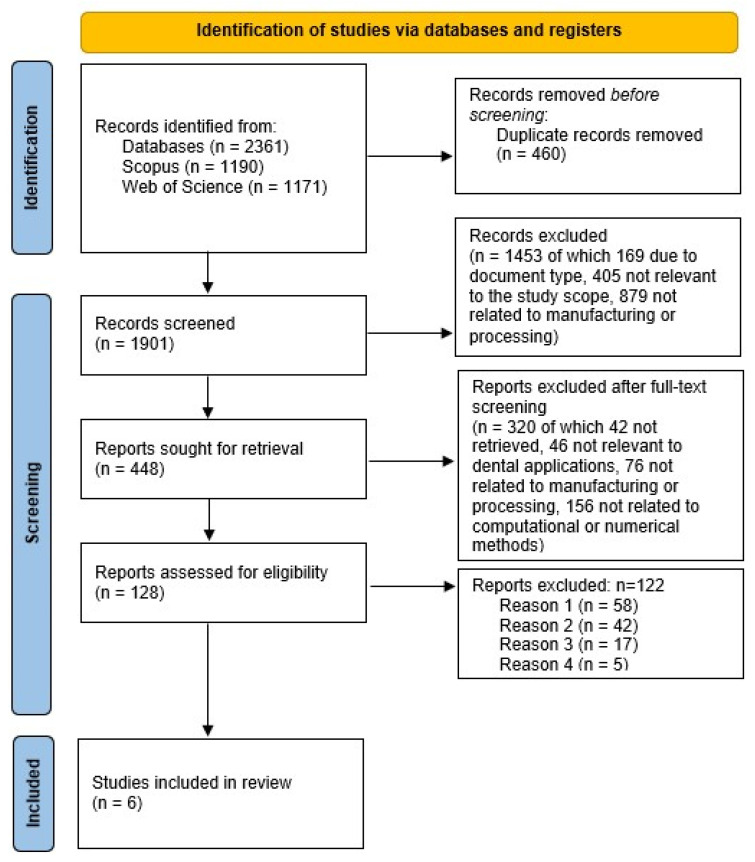
PRISMA flow chart. Reasons for exclusion in the final eligibility stage were defined as follows: Reason 1—studies without mathematical or simulation-based modeling of machining processes; Reason 2—studies focused on biomechanical or structural analysis without a manufacturing-related objective; Reason 3—studies related to additive manufacturing processes (outside subtractive machining); Reason 4—studies related to educational activities, IT (Information Technology) systems, or bibliometric analyses.

The main characteristics and findings of the included studies are summarized in Table 2 and illustrated in the PRISMA flow diagram (see Figure 2).

Table 3 presents a methodological quality assessment of the six studies included in the final qualitative synthesis. The assessment focuses on the validation or evaluation approach, sample or model basis, modeling reliability considerations, and the main limitations of each study. This additional evaluation was included to support a more transparent interpretation of the methodological strengths and constraints of the analyzed modeling approaches.

## 4. Discussion

The objective of this discussion is to critically analyze the current state of mathematical and computational approaches applied in dental bioengineering, with a particular focus on their role in manufacturing and biomaterials processing. The discussion is structured according to the main methodological categories of the included models to compare their contributions, limitations, and applicability across dental biomaterials processing and machining-related technologies. Although the number of included studies is limited, the identified works provide a representative overview of the diverse methodologies currently employed in this field.

Rather than forming a unified modeling framework, the analyzed studies reflect a heterogeneous set of computational strategies, ranging from numerical simulations and statistical modeling to algorithmic and simulation-based approaches. This diversity highlights both the multidisciplinary nature of dental bioengineering and the absence of integrated modeling solutions.

Therefore, this discussion focuses not only on the individual contributions of the selected studies but also on their methodological limitations, lack of integration, and the resulting research gaps. Particular attention is given to the extent to which these approaches enable predictive modeling, process optimization, and practical applicability in dental manufacturing and clinical contexts. In addition to the systematically selected studies, supplementary literature was incorporated to provide broader context and support the critical interpretation of the results.

### 4.1. Mathematical and Computational Approaches in Dental Process Modeling

In this review, numerical, statistical, and algorithmic methods are considered according to their primary modelling objectives: numerical methods are mainly used to evaluate mechanical and thermal responses, statistical methods to quantify relationships between process parameters and output variables, and algorithmic methods to optimize geometry-dependent machining behaviour and process efficiency. The implementation of advanced mathematical and computational methods in dental bioengineering represents a significant shift from purely empirical approaches toward systematic analysis and partially predictive control of technological processes. However, the analysis of the included studies indicates that these approaches do not form a unified modeling framework but rather represent a set of heterogeneous computational strategies addressing different aspects of dental manufacturing processes and biomaterial behavior. Across the included studies, the most frequently considered machining-related parameters included drilling force, rotational speed, drill diameter, feed rate, material removal rate, grinding forces, temperature rise, surface roughness, and machining time, while the main optimization techniques involved DoE, RSM, ANOVA-based evaluation, feed-rate optimization, and geometry-based process modeling.

The mathematical basis of the reviewed approaches can be illustrated through representative formulations corresponding to the main methodological categories discussed in this review. For finite element analysis, the structural response is commonly described by the following equilibrium equation [34]:

(1) K·u=F,

where *K* is the stiffness matrix, *u* is the displacement vector, and *F* is the load vector. Thermal effects during material removal can be generally described by the heat conduction equation [25,33]:

(2) $$\rho \cdot c_{p}\cdot \left(\frac{\partial T}{\partial t}\right)=\nabla \cdot \left(k\cdot \nabla T\right)+\dot{q},$$

where ρ is density, $c_{p}$ is specific heat capacity, *T* is temperature, *t* is time, *k* is thermal conductivity, and $\dot{q}$ represents heat generation during material removal. For response surface methodology, a typical second-order model can be written as [33,36]:

(3) $$Y=\beta_{0}+\sum_{i=1}^{n}\beta_{i}x_{i}+\sum_{i=1}^{n}\beta_{ii}x_{i}^{2}+\sum_{i<j}^{n}\beta_{ij}x_{i}x_{j}+\varepsilon ,$$

where *Y* is the response variable, $x_{i}$ and $x_{j}$ are input process parameters, $\beta_{0}$, $\beta_{i}$, $\beta_{ii}$, and $\beta_{ij}$ are regression coefficients, and ε is the residual error. While material removal rate in algorithm-based machining models can be expressed as [36,37]:

(4) $$MRR=V_{removed}/t_{m},$$

where MRR is the material removal rate, $V_{removed}$ is the removed material volume, and $t_{m}$ is the machining time. The equations above are intended to illustrate the general mathematical principles underlying the reviewed numerical, statistical, and algorithmic approaches. Specific model formulations may differ depending on the analyzed biomaterial, machining process, boundary conditions, input parameters, and optimization objective.

Although the reviewed studies apply different modeling approaches, these methods can be interpreted within a common process–model–output framework. In this framework, material properties, component geometry, and machining parameters represent the main input variables. Numerical models, such as finite element analysis or thermal modeling, are primarily used to predict mechanical or thermal responses. Statistical models, including design of experiments and response surface methodology, describe the relationship between selected process parameters and measurable outputs. Algorithm-based approaches further use these relationships to support process optimization, for example, through feed-rate adjustment, machining-time reduction, or material removal rate prediction. Therefore, the individual modeling approaches are not isolated techniques, but complementary tools that connect biomaterial characteristics, manufacturing conditions, process responses, and optimization objectives. Numerical modeling, as demonstrated by Cuddihy et al. (2013) [34], enables detailed analysis of the mechanical behavior of ceramic materials through the combination of NURBS-based geometric modeling and finite element analysis (FEA). This approach provides high accuracy in stress evaluation and allows for the comparison of different cavity geometries. Similar FEA-based approaches are widely used to investigate stress distribution, thread design, and material behavior in dental implants [39,40]. Nevertheless, their primary limitation lies in the fact that they do not address the machining process itself, but rather the resulting mechanical response of the material. Consequently, these models lack direct linkage to real machining parameters such as cutting forces, feed rates, or thermal effects.

In contrast, statistical modeling approaches, employed by Bogovič et al. (2015) [33] and Mansour et al. (2025) [36], establish a direct relationship between process parameters and output variables. These studies utilize design of experiments (DoE) and response surface methodology (RSM) to develop empirically derived models describing the influence of input parameters on outcomes such as temperature, cutting forces, and surface roughness. While these models provide practical value for process optimization, their empirical nature limits their generalizability beyond the specific experimental conditions under which they were developed.

Algorithmic approaches, as presented by Nam and Kim (2019) [37], focus on geometric and computational modeling of the machining process, particularly in predicting the material removal rate (MRR) and optimizing feed rate during CNC machining of dental crowns. This approach enables improved efficiency and reduced machining time, representing a step toward adaptive process control. However, it is primarily based on geometric relationships and does not incorporate complex physical phenomena such as thermal or mechanical interactions during machining. More advanced algorithmic frameworks, including surrogate modeling and resonance-based prediction methods [41], as well as mathematical models for estimating resonance frequency and micro-displacement based on multi-degree-of-freedom systems [42], and multi-criteria decision-making approaches [43], demonstrate the potential for extending these models toward more sophisticated optimization strategies.

Simulation systems based on human–machine interaction, such as the BoneNavi system described by Ohtani et al. (2009) [38], extend computational approaches into the domain of surgical planning and execution. These systems utilize virtual modeling combined with haptic feedback to simulate drilling processes and improve procedural accuracy. Despite their practical relevance, they do not constitute fully predictive mathematical models of manufacturing processes but rather serve as supportive simulation tools without direct optimization capabilities of technological parameters.

### 4.2. Integration of Geometric Design and Functional Biomaterial Properties

In addition to modeling manufacturing processes, some studies emphasize the importance of linking technological design with functional properties of biomaterials. This perspective is represented by the work of Dantas et al. (2020) [35], which focuses on the design of zirconia surface topographies using CAD/CAM technologies to enhance hydrophilicity and capillary behavior, thereby improving osseointegration.

Machining-induced surface modifications may also have important biological implications, as changes in surface roughness, micro-channel geometry, wettability, and capillary behavior can influence early biological events at the implant–tissue interface. Machined micro- and nano-scale surface features may affect protein adsorption, cell adhesion, osteoblast activity, vascularization, bacterial colonization, and ultimately osseointegration. However, the reviewed studies show that this relationship remains insufficiently integrated into computational modeling frameworks. Most machining-related models focus primarily on process efficiency, mechanical response, thermal effects, or geometrical accuracy, while the biological consequences of machining-induced surface changes are rarely modeled directly. Future studies should therefore link machining parameters and surface integrity descriptors with biological outcomes to improve the translational relevance of dental biomaterial processing models [35].

Recent studies further extend this concept by incorporating advanced structural designs such as functionally graded materials, porous architectures, and biomimetic lattice structures. For instance, Voronoi-based trabecular structures and gyroid TPMS lattices have been shown to significantly improve stress distribution and load transfer efficiency [44], while porosity-gradient scaffolds enhance mechanical stimuli relevant for bone regeneration. Similarly, topology optimization approaches enable improved fatigue performance and structural efficiency in implant design [45], while functionally graded surfaces can be optimized to enhance both mechanical and biological performance [46].

Unlike the other included studies, these approaches do not model the machining process itself but rather address geometric design and subsequent functional performance of the material. Their inclusion highlights the growing importance of integrating design and biological functionality into dental biomaterials. At the same time, they underscore the absence of a comprehensive modeling framework capable of simultaneously linking surface design, manufacturing processes, and biological responses.

### 4.3. Limitations of Current Approaches and Research Gaps

The critical synthesis of the analyzed studies reveals a significant fragmentation in current research. Individual studies tend to focus on isolated aspects such as mechanical behavior, thermal effects, geometric modeling, or process optimization, while lacking systematic integration.

The interpretation and direct comparison of the reviewed studies are limited by the strong interaction between processing parameters, machining conditions, design modifications, modeling assumptions, and material properties. Changes in machining parameters, such as rotational speed, feed rate, drilling force, grinding force, or machining time, may directly influence temperature rise, material removal rate, surface roughness, dimensional accuracy, and the risk of thermally or mechanically induced damage. At the same time, design-related factors, including component geometry, micro-channel architecture, implant shape, or crown configuration, affect stress distribution, tool engagement, and local heat generation. Material-specific properties, such as hardness, brittleness, thermal conductivity, elastic modulus, and fracture resistance, further determine how a biomaterial responds to cutting, drilling, grinding, or CAD/CAM-based surface modification. Therefore, differences in processing conditions cannot be interpreted independently from design and material characteristics, which represents an important challenge for comparing modeling results across different dental biomaterials and manufacturing strategies.

A major limitation lies in the fact that most models are either empirical or partially deterministic, without a comprehensive physics-based representation of the underlying processes. This restricts their predictive capability, particularly outside the conditions under which they were originally developed. Furthermore, there is a lack of integration between computational modeling and real-world manufacturing or clinical processes, particularly in the context of adaptive, real-time process control.

This limitation is further evident in the dominance of biomechanical modeling approaches, which focus primarily on implant–bone interaction rather than on manufacturing processes. Numerous studies apply FEA to analyze porosity effects, stress distribution, and structural optimization [47,48,49], yet these approaches rarely incorporate machining-related parameters or process dynamics.

Another important limitation is the common assumption of material homogeneity and isotropy, which does not reflect the complex and heterogeneous nature of biological tissues, especially bone. This simplification can lead to discrepancies between simulated and actual outcomes, thereby reducing the reliability of predictive models in clinical applications.

### 4.4. Future Research Directions

Based on the identified limitations, future research in dental bioengineering should focus on the development of integrated computational frameworks capable of combining different modeling approaches into comprehensive systems. A key direction is the development of multiphysics models that simultaneously account for mechanical, thermal, and geometric aspects of machining processes.

From a manufacturing perspective, future implant production should more explicitly link material selection, surface design, machining parameters, and computational prediction [27,31]. For dental implant manufacturing, this means that material-specific properties, such as the behavior of titanium, zirconia, ceramics, or porous biomimetic structures, should be considered together with process parameters including feed rate, cutting or drilling forces, rotational speed, temperature rise, surface roughness, and material removal rate [35,36,37]. Computational models should therefore support not only biomechanical evaluation after implant design but also the optimization of manufacturing conditions to improve dimensional accuracy, surface quality, thermal safety, and long-term functional performance [41].

Significant potential also lies in the implementation of digital twins, which enable real-time integration of numerical models with sensor-based data from manufacturing or surgical processes [9,10,11]. Such systems could support adaptive process control and improve the precision and reliability of dental procedures [30,31,32].

Furthermore, future research should emphasize personalized modeling approaches that incorporate patient-specific anatomical and material characteristics [12,13]. The integration of geometric design, manufacturing processes, and biological response into a unified framework represents a major challenge but also a necessary step toward advancing predictive and optimized dental bioengineering [19,20].

## 5. Conclusions

This systematic review identified a limited number of studies that explicitly connect dental biomaterials processing or machining with mathematical, statistical, or computational modeling. The included studies addressed PMMA bone analogs, ceramic dental crowns, zirconia dental implants and crowns, alumina ceramics, and bone models used for implant drilling simulation. The analyzed processes included implant drilling, grinding, CNC milling, CAD/CAM-based surface processing, and guided drilling simulation.

The reviewed approaches were mainly based on finite element analysis, NURBS-based geometric modeling, response surface methodology, ANOVA-supported statistical evaluation, algorithm-based material removal prediction, and CAD/CAM-supported simulation systems. These methods were used to evaluate or optimize specific parameters, including drilling force, rotational speed, drill diameter, feed rate, material removal rate, grinding forces, temperature rise, surface roughness, machining time, dimensional accuracy, and stress distribution.

The main finding is that current modeling approaches remain highly process-specific and fragmented. Most studies focus on selected mechanical, thermal, geometrical, or process-related outputs rather than on integrated prediction of biomaterial behavior during manufacturing. Simplified assumptions regarding material homogeneity and isotropy may further limit the reliability of simulations under clinically relevant conditions.

Future research should be focused on material-specific and process-specific predictive models that link biomaterial properties, component geometry, machining parameters, surface integrity, and functional performance. The integration of multiphysics modeling, digital twins, adaptive process control, and patient-specific modeling may support more reliable and optimized dental biomaterial processing.

## Figures and Tables

**Table 1:** Articles’ Screening Strategy. The asterisk (*) indicates wildcard/truncation in the database search.

Search Strategy Component	Description
**Keywords (A)**	dental bioengineering; dental biomaterials; oral biomaterials; dentistry; prosthodontics; implantology
**Keywords (B)**	machining; manufacturing; processing; milling; drilling; grinding; CAD/CAM; additive manufacturing; 3D printing
**Keywords (C)**	mathematical model *; computational model *; simulation; finite element analysis; FEA; FEM; optimization; numerical analysis; algorithm *; predictive model *
**Boolean Indicators**	(A) and (B) and (C)
**Timespan**	from the earliest available records to [04.2026]
**Electronic Databases**	Scopus; Web of Science Core Collection (WoS)

**Table 2:** Summary of Selected Studies in Dental Bioengineering and Biomaterials Processing.

Authors (Year)	Material/ System	Process Type	Applied Method	Modelling Approach	Objective	Key Contribution
Bogovič et al. (2015) [33]	Bone analog material (PMMA—Polymethyl Methacrylate), dental implant system	Drilling (implant site preparation)	Design of Experiments (DoE), Response Surface Methodology (RSM)	Mathematical and statistical modeling	To model temperature evolution during implant drilling	Identification and optimization of key process parameters (drilling force, rotational speed, drill diameter) to minimize thermal load
Cuddihy et al. (2013) [34]	Ceramic dental crowns	Cavity preparation (drilling/grinding)	Finite Element Analysis (FEA), NURBS (Non-Uniform Rational B-Splines)-based geometric modeling	Numerical simulation	To simulate endodontic access cavity preparation in ceramic crowns	Development of a 3D model based on CT (Computed Tomography) data and FEM analysis of stress distribution for geometry optimization
Dantas et al. (2020) [35]	Zirconia dental implants	Surface processing (CAD/CAM machining)	Experimental design, geometric surface modeling	Engineering design and process-oriented optimization	To optimize surface topography of zirconia implants	Design of microstructured surfaces (microchannels) improving wettability and capillary behavior
Mansour et al. (2025) [36]	Alumina ceramics (relevant to dental applications)	Ultrasonic-assisted grinding	Design of Experiments (DoE), Response Surface Methodology (RSM), ANOVA (Analysis of Variance)	Statistical and numerical modeling	To optimize grinding process parameters	Multi-objective optimization of material removal rate, grinding forces, and surface roughness
Nam & Kim (2019) [37]	Zirconia dental crowns	CNC (Computer Numerical Control) milling/grinding	Mathematical modeling, algorithm development	Algorithm-based modeling	To reduce machining time via material removal rate optimization	Development of a predictive model for material removal rate (MRR—Material Removal Rate) and feed rate optimization for complex geometries
Ohtani et al. (2009) [38]	Pig bone model, dental implant system	Drilling (implant placement)	CAD/CAM-based simulation, haptic system, experimental validation	Computational simulation with experimental validation	To evaluate drilling accuracy using a computer-assisted system	Validation of drilling precision and improvement of implant placement accuracy using the BoneNavi simulation system

**Table 3:** Methodological quality assessment of the included studies.

Authors (Year)	Validation or Assessment Approach	Sample/Model Basis	Modeling Reliability Considerations	Main Limitations
Bogovič et al. (2015) [33]	Experimental drilling simulation with temperature measurement; DoE/RSM and experimental confirmation of optimized parameters.	PMMA test specimens used as bone analogs; drilling force, drill diameter, and drilling speed.	Reliable within the tested parameter ranges and controlled experimental setup.	Limited transferability to clinical bone due to the use of PMMA and simplified experimental conditions.
Cuddihy et al. (2013) [34]	CT-based reconstruction, NURBS geometric modeling, and FEA comparison of three access cavity configurations.	Ceramic crown model reconstructed from CT data.	Dependent on geometry reconstruction, loading, boundary conditions, and material property assumptions.	Focused on mechanical response after virtual cavity preparation, not on direct machining parameter optimization.
Dantas et al. (2020) [35]	CAD/CAM manufacturing and experimental assessment of zirconia micro-channel surfaces.	3Y-TZP zirconia specimens with machined micro-channel geometries.	Suitable for evaluating surface design, wettability, capillarity, and surface morphology.	Not a predictive machining model; limited direct biological validation of the proposed surface design.
Mansour et al. (2025) [36]	RSM/statistical modeling and optimization of MRR, grinding forces, and surface roughness.	Alumina ceramics under ultrasonic vibration-assisted end grinding.	Reliable mainly within the investigated material, setup, and process parameter limits.	Empirical and process-specific; limited generalizability to other materials or grinding conditions.
Nam and Kim (2019) [37]	Algorithm-based MRR prediction and machining-time simulation for feed-rate adjustment.	Zirconia dental crown geometry in CNC milling/grinding.	Dependent on the accuracy of geometric engagement modeling and simulation assumptions.	Thermal effects, cutting forces, tool wear, and surface integrity were not explicitly addressed as the central focus.
Ohtani et al. (2009) [38]	Accuracy verification of the BoneNavi CAD/CAM-based simulation and haptic drilling system.	Pig bone model used for implant drilling simulation.	Relevant for evaluating guided drilling accuracy and surgical simulation precision.	Focused on drilling accuracy rather than predictive modeling or optimization of machining parameters.

## Data Availability

No new data were created or analyzed in this study. Data sharing is not applicable to this article.

## Supplementary Materials

The following supporting information can be downloaded at: https://www.mdpi.com/article/10.3390/biomimetics11070448/s1, (PRISMA 2020 checklist) [50].

## Abbreviations

The following abbreviations are used in this manuscript:

3D	Three-dimensional
ANOVA	Analysis of Variance
CAD	Computer-Aided Design
CAM	Computer-Aided Manufacturing
CNC	Computer Numerical Control
CT	Computed Tomography
DOE	Design of Experiments
FEA	Finite Element Analysis
FEM	Finite Element Method
FGMs	Functionally Graded Materials
IT	Information Technology
MRR	Material Removal Rate
NURBS	Non-Uniform Rational B-Splines
PMMA	Polymethyl Methacrylate
PRISMA	Preferred Reporting Items for Systematic Reviews and Meta-Analyses
RSM	Response Surface Methodology
TPMS	Triply Periodic Minimal Surfaces
WoS	Web of Science Core Collection
